# Inactivity and Skeletal Muscle Metabolism: A Vicious Cycle in Old Age

**DOI:** 10.3390/ijms21020592

**Published:** 2020-01-16

**Authors:** Elena Rezuş, Alexandra Burlui, Anca Cardoneanu, Ciprian Rezuş, Cătălin Codreanu, Mirela Pârvu, Gabriela Rusu Zota, Bogdan Ionel Tamba

**Affiliations:** 1 Department of Rheumatology and Physiotherapy, “Grigore T. Popa” University of Medicine and Pharmacy, 700115 Iaşi, Romania; elena.rezus@umfiasi.ro (E.R.); anca.cardoneanu@umfiasi.ro (A.C.); 2 Department of Internal Medicine, “Grigore T. Popa” University of Medicine and Pharmacy, 700115 Iaşi, Romania; ciprian.rezus@umfiasi.ro; 3 Center for Rheumatic Diseases, “Carol Davila” University of Medicine and Pharmacy, 050474 Bucharest, Romania; catalin.codreanu@reumatologiedrstoia.ro; 4 Department of Rheumatology and Physiotherapy,“George Emil Palade” University of Medicine, Pharmacy, Science and Technology, 540139 Târgu Mureş, Romania; mirela.parvu@umfst.ro; 5 Department of Pharmacology, Clinical Pharmacology and Algesiology, “Grigore T. Popa” University of Medicine and Pharmacy, 700115 Iaşi, Romania; rusu.i.gabriela@umfiasi.ro; 6 Advanced Center for Research and Development in Experimental Medicine (CEMEX), “Grigore T. Popa” University of Medicine and Pharmacy, 700454 Iaşi, Romania; bogdan.tamba@umfiasi.ro

**Keywords:** aging, sarcopenia, muscle mitochondria, catabolism, anabolism, inflammation, adipokine, myokine, sedentary lifestyle, exercise

## Abstract

Aging is an inevitable and gradually progressive process affecting all organs and systems. The musculoskeletal system makes no exception, elderly exhibit an increased risk of sarcopenia (low muscle mass),dynapenia (declining muscle strength), and subsequent disability. Whereas in recent years the subject of skeletal muscle metabolic decline in the elderly has been gathering interest amongst researchers, as well as medical professionals, there are many challenges yet to be solved in order to counteract the effects of aging on muscle function efficiently. Noteworthy, it has been shown that aging individuals exhibit a decline in skeletal muscle metabolism, a phenomenon which may be linked to a number of predisposing (risk) factors such as telomere attrition, epigenetic changes, mitochondrial dysfunction, sedentary behavior (leading to body composition alterations), age-related low-grade systemic inflammation (inflammaging), hormonal imbalance, as well as a hypoproteic diet (unable to counterbalance the repercussions of the age-related increase in skeletal muscle catabolism). The present review aims to discuss the relationship between old age and muscle wasting in an effort to highlight the modifications in skeletal muscle metabolism associated with aging and physical activity.

## 1. Introduction

Aging is an inevitable process affecting all organs and systems [1]. The musculoskeletal system makes no exception, the elderly exhibit an increased risk of degenerative joint disease, muscle loss (sarcopenia), declining muscle strength (dynapenia), and subsequent disability [2]. Medicine has seen notable progress in the last decades, leading to a dramatic increase in life expectancy, especially in developed countries [3]. It has been stated that by the end of the 21st century, median ages will ascend significantly [4]. Consequently, the economic burden of age-related disorders is increasing and may imply a stringent need for more numerous specialized health care professionals in the upcoming years [5]. Global estimates anticipate that by 2050 a particularly large proportion of the general population will be over 60 years of age, which makes the issue of improving the management of aging-associated disorders of paramount importance [4,6].

Skeletal muscle assures locomotion as well as certain important aspects of metabolic homeostasis, such as glucose uptake and fatty acid oxidation [7]. Among other issues, the subject of age-related muscle impairment has been brought into focus by the growing number of elderly individuals requiring medical assistance [4,5,6] and the important functional hindrance linked to musculoskeletal system alterations in old age [8,9,10,11,12].

From debilitating comorbidities [13], inactivity and immobilization [14] to aging-associated neurological and immune abnormalities, hormonal imbalance, oxidative stress, as well as poor nutrition, a plethora of factors contribute to the development of sarcopenia in the elderly. Moreover, it has been shown that aging triggers important changes at the level of skeletal muscle metabolism; therefore, enticing the appearance of clinically relevant disruptions in the global metabolic homeostasis [15,16].

The present review aims to discuss the relationship between old age and muscle wasting in an effort to highlight the changes in skeletal muscle metabolism associated with aging and physical activity.

## 2. Senescence and Human Skeletal Muscles

In recent years, the subject of cell senescence (derived from the Latin word senescere portraying age-related decline or waning) has been studied intensely [16]. In the 1950s, one of the first theories regarding cell senescence was proposed describing a central role for reactive oxygen species (ROS) in the process of aging [17]. The presence of free radicals may induce telomere shortening (the latter structures being sensitive to excessive ROS levels); thus, promoting the appearance of DNA alterations and contributing to cellular aging. In vitro studies presented replicative senescence as the cultured cells’ failure to divide after 30–40 doublings (known as the Hayflick limit) while also exhibiting telomere length attrition and the formation of age-related heterochromatin [16,18]. Moreover, it has been proven that skin fibroblasts isolated from older individuals present with similar alterations of their genetic material [19].

Skeletal muscle cells demonstrate longer telomeres compared to leukocytes. However, telomere shortening has been shown to progress at similar rates in minimally proliferative tissues (such as skeletal muscle cells and adipocytes) and proliferative cells (such as skin cells and leukocytes) [20]. Nevertheless, telomere length displays notable inter-individual discrepancies with a concomitant "synchrony" across different somatic tissues (a strong correlation between tissues, different subjects presenting either shorter or longer telomeres) in humans, non-human primates, non-primate mammals, and birds [21,22,23,24,25].

It has been reported that the amassing of connective tissue in muscles together with lipid molecule build-up (nonesterified-free fatty acids and triglycerides, as well as the respective metabolites) [26] inside myocytes (skeletal muscle myosteatosis being considered a particular type of ectopic fat depot) may largely contribute to the decline of muscle quality in old age, with marked metabolic consequences [27,28,29].

**Figure 1:**
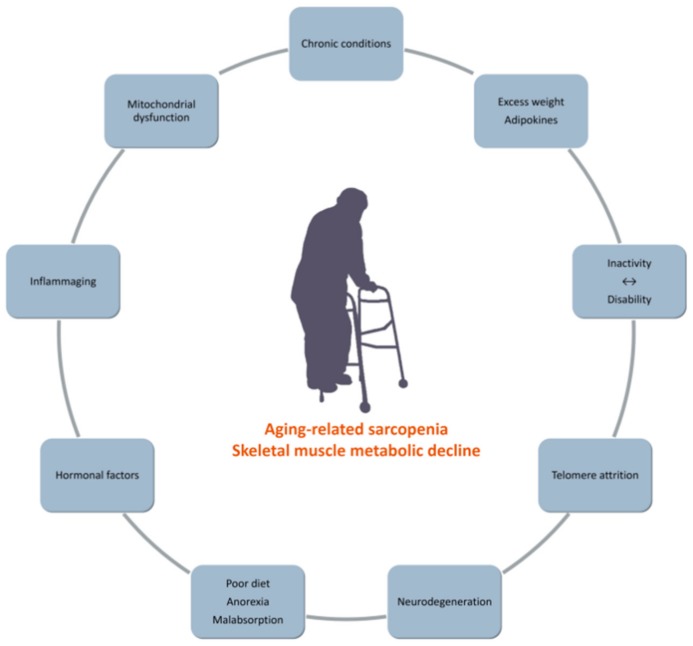
Associated with age-related sarcopenia.

Sarcopenia and dynapenia are highly prevalent in the elderly as well as in chronic conditions (Figure 1) [30,31,32]. From the age of 30, muscle mass decreases by circa 40 percent during a period of 30 years, followed by a more rapid decline in the next decades (up to 40 percent per decade after 60 years of age) [27]. Consequently, muscle mass constitutes just one-quarter of the total body mass in persons in their late 70s [27]. Moreover, it has been stated that sarcopenia could be regarded as a geriatric syndrome [33]. As sarcopenia is known to associate with both hypoplasia (the decline of fiber number) as well as skeletal muscle atrophy (the reduction of fiber size), it demonstrates notable differences from disuse-related changes in skeletal muscle mass which commonly only involve a decrease in fiber size [34].

While not yet fully understood, the pathomechanisms underlying sarcopenia and dynapenia, have become a legitimate subject of research in recent years [35]. Fabbri et al. showed that a higher total fat mass accompanied by a simultaneous loss in lean body mass predicts a more rapid rate of decline in muscle quality (the strength-to-mass ratio defined as the relationship between knee extension strength and thigh muscle cross-sectional area) in persons over 50 years of age during a four-year follow-up period [35]. Nevertheless, the authors did not obtain a significant relationship with weight or body mass index (BMI) [35].

In the elderly, the dynamics of energy production and usage are disrupted [36,37]. This phenomenon yields more severe repercussions in older persons with multiple chronic disorders. In this regard, elderly individuals with varied chronic diseases demonstrate a more increased resting metabolic rate compared to their healthy counterparts while exhibiting lower available energy [36,38]. Ager-related loss of muscle mass is linked to the reprogramming of skeletal muscle metabolism [39]. 

**Figure 2:**
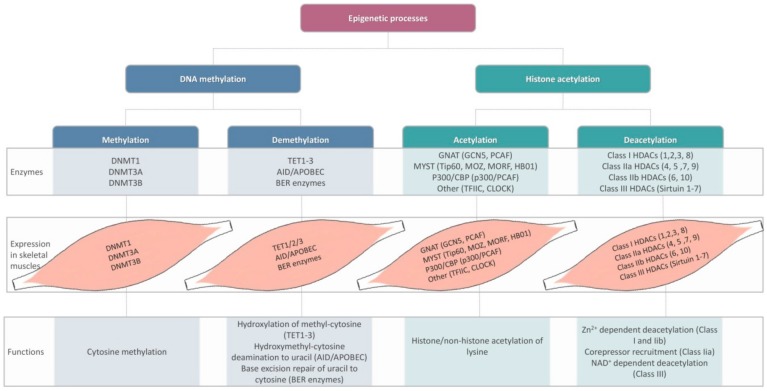
Epigenetic changes, enzymes involved, skeletal muscle expression, and metabolic consequences. DNMT: DNA methyltransferase; TET: ten-eleven translocation; AID: activation-induced cytidine deaminase; APOBEC: apolipoprotein B mRNA editing enzyme component 1; BER: base excision repair; GNAT: Gcn5-related N-acetyltransferase; CBP: Creb-binding protein; HDAC: histone deacetylase (after [40]).

Ranging from DNA methylation/demethylation to histone acetylation/deacetylation processes, a wide variety of epigenetic alterations have been thought to modulate skeletal muscle metabolic activity (Figure 2) [40]. In light of recent research, inhibitors of histone deacetylase (HDAC) superfamilies emerge as potential anti-aging agents in both animal models as well as human subjects [41]. Certain repair mechanisms such as BER (base-excision repair) are believed to maintain genome stability. It was stated that physical exercise could improve BER enzyme activity at the level of skeletal muscles in rodents [42]. However, the mechanisms underlying exercise/inactivity-related epigenetic responses on the development of sarcopenia in elderly human subjects, as well as the potential consequences of skeletal muscle "epi"-memory with respect to physical activity are still a matter of discussion [43,44].

Certain chronic conditions such as type II diabetes mellitus and neuromuscular diseases, as well as age-related loss of muscle mass, have been shown to associate with epigenetic changes, consequently, leading to modifications of skeletal muscle metabolic activity [40]. However, together with other factors such as nutrient availability, exercise may regulate epigenetic response [45]. Moreover, as shown by the bioinformatics meta-analysis performed by Brown, epigenetic responses post-exercise (particularly DNA methylation/demethylation) may greatly depend on age [46].

Endurance exercise has been shown to decrease global DNA methylation in young sedentary individuals. In mice [47] as well as human subjects, endurance exercise lead to modifications of DNA methylation patterns, mainly involving genes related to skeletal muscle differentiation, metabolism, and growth [48]. Drummond et al. found that resistance exercise (combined with amino acid supplementation) was not followed by a skeletal muscle downregulation of miR-1 in aging compared to young individuals [49]. Nevertheless, other studies reported discrepant results [50].

### 2.1. Structural Decline

Motor units within skeletal muscles may be stratified according to the type of myosin dimers displayed by the fibers. Specifically, type I myosin demonstrates an abundance of mitochondria and myoglobin, in this case, ATP deriving from oxidative metabolic processes. Type I myosin characterizes low fatigable motor units in which it is capable of transducing energy over lengthier periods of time at a relatively decreased pace [27]. Contrastingly, myosin type IIx contained within fast fatigable muscle fibers mainly relies on glycogen lysis; therefore, assuring higher amounts of usable energy for shorter time periods [27]. Motor units displaying type IIa myosin are considered intermediate on several levels (velocity of energy transduction, the cross-sectional area size, fiber number). Noteworthy, these fibershave been described, as fast, yet fatigue-resistant [27].

In elderly sarcopenic individuals, type II muscle fibers have been shown to be more prone to atrophy compared to type I predominantly in postural muscles. However, studies report discrepant results with regard to the proportion in which the cross-sectional area of fast fatigable (type II) or slow fatigable fibers (type I) decreases with aging [34]. In this respect, the cross-sectional area size was found to be lower by circa one-quarter to more than half in subjects over 80 years of age compared to young controls. Though less dramatic, a significant decline of the type I fiber cross-sectional area size in the elderly was reported by some studies, but not by others, the subject of slow fatigable fiber atrophy in aging remains a matter of discussion [34]. 

Age-related dysregulations in motor units may translate into significant disability. Nevertheless, an aging-related conversion of slow fatigable fibers into fast fibers, as well as an increment of hybrid fibers, have been observed [27]. It has been stated that neurodegeneration gives rise at least partly to these changes, denervated fibers being recruited by the remaining functional motor units, which have a tendency to gather similar fiber types while simultaneously altering fibers to suit the characteristics of the motor unit [27].

Murgia et al. analyzed the impact of aging on skeletal muscle from a single-fiber proteomic perspective (mass spectrometry-based proteomics) in eight physically active older versus younger adults (widely spaced with respect to age) in an effort to eliminate the important confounding factors that are inactivity and immobilization in the elderly [7]. Numerous glycolytic enzymes displayed a significantly increased expression in older individuals, while an important reduction was observed in fast fibers (type IIx glycolytic fibers) [7]. It has been reported previously that age-associated sarcopenia notably impacts the fast type of skeletal muscle fibers in human subjects, but not the slow-twitch type (type I oxidative fibers) [51].

Vigelsø et al. compared biopsies from the vastus lateralis of endurance-trained middle-aged men to that of controls (untrained), finding higher contents of perilipin 5, endothelial lipase (which was correlated with circulating high-density lipoprotein values, HDL), and mitochondrial complex III–V in trained individuals [52].

The accumulation of certain bioactive lipids such as ceramide and diacylglycerols(DAGs) at the level of skeletal muscles has been linkedto insulin sensitivity in experimental animals and human subjects [53,54]. It has been stated that sarcolemma ceramide (particularly the C18:0 species), as well as sphingolipids, may be inversely associated with insulin sensitivity [54]. Søgaard et al. examined the effects of a six-week high-intensity interval training (HIIT) on DAG and ceramide expression in muscle biopsies of both young and older subjects with excess weight [55]. Muscle ceramide anddiacylglycerol levels were found to be higher in older compared to younger patients, while HIIT diminished C18:0 and saturated ceramides. In addition, exercise prompted the elevation of certain molecules involved in lipid and glucose metabolism (fatty acid-binding protein, adipose triglyceride lipase, glycogen synthase, GLUT4, hexokinase II) [55].

### 2.2. The Aging Mitochondrion

The mitochondrion is a highly dynamic organelle that holds a pivotal role in energy production as well as the release of ROS [56]. Moreover, mitochondria have been shown to coordinate important phenomena such as cell death and signaling, thus playing a more complex part in the regulation of global homeostasis than it was previously thought [57]. Skeletal muscle mitochondrial dysfunction is highly prevalent in advanced age; the latter associating with a decrease in both mitochondrial volume and number, as well as reduced biogenesis [56].

**Figure 3:**
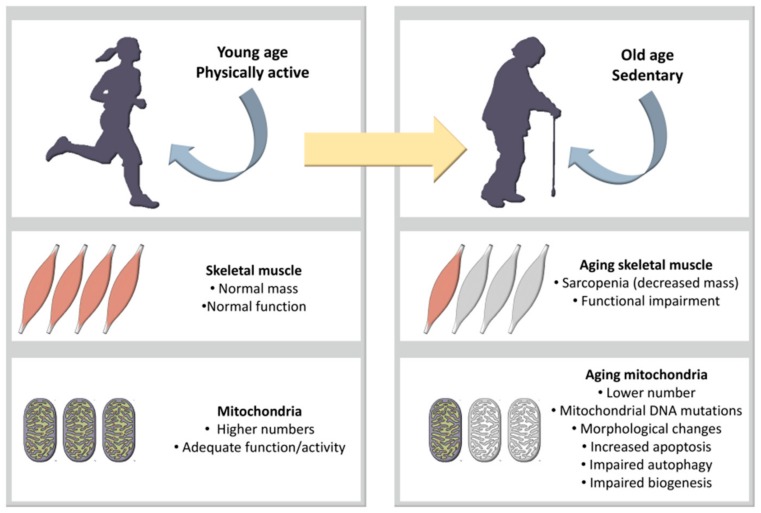
Skeletal muscle mitochondria [56].

Advanced age is associated with remarkable bioenergetic and biochemical changes in skeletal muscle mitochondria (Figure 3) [56]. In this respect, a decrement of enzymatic activity together with notable changes involving oxidative stress and mitochondrial DNA mutations (accompanied by the alteration of oxidative phosphorylation and subsequent mitochondrial dysfunction) have been observed in aging individuals [58,59].

Sirtuin-3 (Sirt3, a member of the sirtuin family) is an NAD+–dependent protein deacetylase exerting regulatory properties on mitochondrial activity. In murine models of diabetes, its expression in skeletal muscle is reduced leading to anomalies in ROS production and insulin signaling [60]. Moreover, Sirt3 has been shown to be associated with aging [61,62,63].

Menshikova et al. investigated the discrepancies in mitochondrial size and content in the skeletal muscles (vastus lateralis percutaneous biopsies) of overweight/obese older individuals undergoing either physical training or calorie restriction [64]. In the exercise group, the authors found a significant increase of muscle mitochondria size and mitochondria membrane content (cardiolipin). Additionally, the activity of the electron transport chain (rotenone-sensitive NADH-oxidase) and the β-oxidation pathway (β- hydroxyacyl CoA dehydrogenase activity) were also enhanced at 16 weeks of training compared to baseline [64]. Calorie restriction only promoted tricarboxylic acid cycle activity (citrate synthase) [64].

## 3. Body Composition and Physical Activity in Old Age

Seeing as there is a growing body of evidence supporting the association between advanced age and body composition alterations, medical professionals are becoming increasingly aware of the challenges posed by aging in the general population [65].

The European Working Group on Sarcopenia in Older People (EWGSOP) published a consensus report advancing diagnosis criteria for sarcopenia. The aforementioned criteria propose the evaluation of the decline in skeletal muscle mass together with strength and physical performance, thus arguing against the separation of sarcopenia from dynapenia [66].

However, other authors defend the concept of a disproportionate decline between muscle mass and strength in old age, with dynapenia possibly progressing more rapidly than the decrease in muscle size [34]. In addition, dynapenia has been thought to describe the functional hindrance derived from both muscle- and neural-related factors (the neuromuscular apparatus as a whole) [67,68]. Nevertheless, EWGSOP presented the means for stratification of sarcopenic patients according to the severity of the changes identified, defining presarcopenia as the decrease in muscle mass alone.Sarcopenia was described as a decrease in muscle mass coexisting with either diminished muscle strength or low physical performance. Furthermore, EWGSOP defined severe sarcopenia as the association between the three parameters [66]. While the concomitance of skeletal muscle mass decline and muscle-related functional impairment is regarded as clinically relevant by authors irrespective of their position towards the temporal disassociation between the twoaforementioned aspects of the aging process, the concept of dynapenia (analyzed by itself) remains a subject of controversy and discussion [30,66].

Part of the data resulting from the Health, Aging, and Body Composition Study (Health, ABC) [69] included protein intake and the loss of lean mass (LM) and non-bone appendicular lean mass (aLM) over a three-year follow-up period in a cohort of non-frail community-dwelling older adults (2066 men and women between 70–79 years of age). The daily dietary protein intake was estimated using a 108-item food frequency questionnaire (FFQ), while subjects’ body composition was evaluated by dual X-Ray absorptiometry (DXA) [69]. The study determined that subjects in the lowest protein quintile suffered a loss of lean body mass circa 40% greater (43% for LM and 39% aLM) than the highest protein quintile over three years, thus emphasizing the role of nutrition in the preservation of muscle mass in the elderly [69].

In older individuals, the balance between protein synthesis and breakdown may be hindered. Increased muscle catabolism and the reduction of skeletal muscle mass both characterize old age and frailty. It has been reported that frailty enhances the development of aging-related disruptions of protein metabolism [70]. The lack of dietary proteins is a potential factor involved in the decrease of muscle protein synthesis in the elderly. Studies showed that aminoacid supplementation might demonstrate a beneficial effect on muscle protein synthesis in old, as well as young subjects [71]. However, some authors argue that supplementation may not be sufficient in order to induce significant changes in muscle catabolism [72].

Dietary protein intake has often been shown to be below the recommended daily allowance in both men and women. In addition, research focusing on dietary intake for efficient stimulation of protein synthesis concluded that the optimal daily consumption falls within 1.2 and 1.5 g/kg [72,73]. Currently, it is believed that the recommended daily allowance (0.8 g/kg) is too low to assure the conservation of muscle mass in aging subjects. In frail elderly women, higher dietary protein intake has been shown to boost skeletal muscle metabolism [74].

### 3.1. Skeletal Muscle and White Adipose Tissue in Old Age: AComplex Relationship

Whereas middle age is characterized by weight gain in both men and women, elderly individuals experience a decrease in body mass, which differs according to gender. In this regard, it has been found that weight loss in old age occurs earlier in men compared to women [75]. In contrast, the amount of visceral fat follows an ascending curve during aging, with abdominal circumference demonstrating growing values in the elderly [36].

As of recent discoveries, white adipose tissue is no longer considered only a mean of storing excess energy, its active and dynamic secretory activity being described by numerous studies [76,77]. In this respect, research showed that the various protein and peptide molecules released by adipocytes play an important role in regulating global homeostasis. Over 100 of these compounds called adipokines (also referred to as adipocytokines) have been described so far [78]. Leptin, the first adipokine described in the literature, plays a major role in coordinating food intake through its action on the hypothalamus as an anorexigenic hormone [79]. Specific receptors for leptin have been identified in the kidneys, lungs, adrenal glands, and skeletal muscles, suggesting its involvement in the modulation of the aforementioned tissue/organ activity [80,81,82].

**Figure 4:**
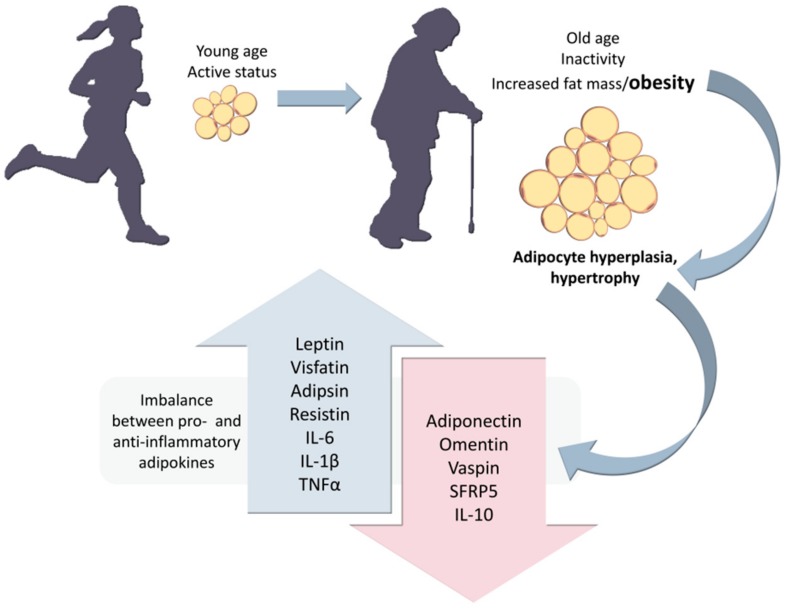
Size-dependent imbalance between pro-inflammatory adipokines (such as leptin, visfatin, adipsin, resistin, interleukin-6 (IL-6), IL-1β, and tumor necrosis factor α (TNFα)) and anti-inflammatory adipokines (adiponectin, omentin, visceral adipose tissue-derived serine protease inhibitor (vaspin), secreted frizzled-related protein 5 (SFRP5), and IL-10) [79,80,85].

Obesity is frequent among older sedentary individuals and has been shown to associate with both adipocyte hyperplasia and hypertrophy, thus triggering an imbalance between pro- and anti-inflammatory adipokine release (Figure 4) [76,78,83,84].

In centenarians, enhanced leptin levels have been linked to increased longevity. Moreover, sarcopenic individuals exhibit lower leptin titers [84]. Seeing as leptin receptors are abundant in skeletal muscles, it has been stated that leptin may regulate metabolic activity at this level [85,86]. Nonetheless, while classically considered a mainly pro-inflammatory adipokine, leptin may also be released by skeletal muscles, thus becoming an adipomyokine. When comparing skeletal muscle to adipose tissue, the latter releases only a somewhat larger amount of leptin (per unit mass) [87].

Several pathological conditions are associated with sedentary behavior, sarcopenia, and increased fat mass. Leptin levels have been shown to be increased in patients with spinal cord injuries [85]. Yet sarcopenic older subject demonstrates lower leptin concentrations [84]. In vitro studies provided evidence regarding the impact of leptin concentrations on muscle metabolism. In cultured differentiated cultured muscle cells (C1C12 myotubes), higher leptin concentrations promptly promoted the expression of myokine and energy metabolism genes; therefore, influencing nutrient partition as well as oxidative processes [88]. In this respect, Nozhenko et al. found that peroxisome proliferator-activated receptor γ coactivator-1α (PGC-1α), uncoupling protein 3 (UCP3), muscle carnitine palmitoyltransferase 1 (mCPT1), insulin receptor (InsR), interleukin-6 (IL-6), IL-15, and leptin receptor (OB-Rb) [88].

The amount of intermuscular fat located in the lower extremities may impact skeletal muscle quality and performance in certain populations such as older subjects. Moreover, it has been reported that intermuscular adipocytes secrete inflammatory mediators, thus creating an inflammatory microenvironment that is partly responsible for the appearance of alterations in the local blood supply [35]. In this context, skeletal muscle fibers suffer several metabolic changes, such as increased lipolysis followed by the accumulation of glucose within the tissue. Nevertheless, the latter phenomenon contributes to the development of insulin resistance. The link between the secretory activity of intermuscular white adipose tissue and insulin resistance has long been investigated, the currently available scientific data suggesting that obesity is not the sole culprit in the appearance of metabolic anomalies in old age. In addition to intermuscular adipose tissue, collagen deposition may also negatively impact muscle quality [35]. 

By promoting the accumulation of inflammatory cells within skeletal muscles and the simultaneous boosting of pro-inflammatory responses at the level of intermuscular fat, obesity ultimately leads to muscle inflammation. These phenomena incite the appearance of notable anomalies in myocyte metabolism [89]. Moreover, myocyte differentiation and satellite cell (also known as muscle stem cells) activity may be impaired by muscle fat depots. Together with aging, obesity has been shown to sustain a chronic pro-inflammatory state while also hinders muscle regeneration. Furthermore, this condition is frequently accompanied by sarcopenia in elderly individuals (sarcopenic obesity) [89].

### 3.2. Exercise Versus Inactivity

Apart from its role in assuring the erect posture in humans as well as locomotion, skeletal muscle tissue has long been proven to regulate global glucose metabolism [90,91]. Research indicates that skeletal muscle metabolic phenotypes may be partly inherited. In murine studies, mitochondrial dysfunction, impaired insulin signaling, and reduced glucose transporter 4 (GLUT4) expression in skeletal muscles have been observed in the offsprings born to obese mothers [92,93]. However, numerous other factors drive significant changes in skeletal muscle metabolism [40]. In this respect, physical exercise ameliorates skeletal muscle function by improving metabolic processes [94].

Relative to rest periods, the fractional production rate of myofibrillar proteins may be raised by exercise in sedentary older men, with better results at 24- and 48-h post-exercise in patients undergoing resistance training compared to HIIT [95]. Nevertheless, in the study performed by Bell et al. HIIT constituted the single exercise regimen, which resulted in the bolstering of sarcoplasmic protein fractional synthetic rate one-day post-exercise [95].

In recent studies, skeletal muscle emerges as an important secretory organ with a plethora of roles in the control of global homeostasis. Myokines are thought to be mainly secreted by muscles but may also be released by hepatic, pancreatic, and adipose tissues [91]. These peptides trigger complex metabolic changes through endocrine, paracrine, as well as autocrine signaling [91].

Apelin is an adipomyokine whose release may be upregulated by endurance training in individuals with excess weight [96]. It has been stated that apelinomimetic agents (or an agonist of the APJ receptor which is a G protein-bound receptor expressed in numerous tissues) could be used to improve aging-related skeletal muscle dysfunction [97]. In murine models of insulin resistance, apelin treatment exerted an important influence on the activity of skeletal muscle mitochondria. In this regard, apelin promoted biogenesis, glucose transport, complete fatty acid oxidation, and oxidative capacity [97].

Huh et al. found higher irisin values in younger versus older research subjects, studies reporting an inverse relationship between age and circulating irisin titers [98]. Irisin is a recently described myokine [99] that has been proposed as an “exercise hormone” (initially thought of as an exercise-inducible signaling molecule) resulting from the proteolytic cleavage of membrane fibronectin type III domain-containing protein 5 (FNDC5) [100]. It has been stated that white adipose cells may go through a “browning” process under the influence of irisin, the result being the appearance of beige/brite adipocytes with particular metabolic characteristics [99,101,102].However, the numerous discrepancies in published data regarding both the detection methods as well as the potential relevance of irisinin humans lead to significant controversy [103]. One of the main concerns raised by authors involved the precision of the Enzyme-Linked Immunosorbent Assay (ELISA) kits used to estimate irisin levels [103,104]. Four commercially available polyclonal antibody kits for irisin demonstrated marked cross-reactivity with non-specific proteins when analyzed by Western blotting. Albrecht et al. found this to be true in both human sera as well as animal samples [104].

In vitro research indicated that primary human skeletal muscle cells (HMSCs) treated with irisin suffer alterations in the expression of metabolic genes. In this respect, the upregulation of such genes involved in glucose metabolism as hexokinase 2 (HK2) and glucose transporter 4 (GLUT4) was observed as early as si hours post-treatment by Huh et al. [105]. The upregulation of genes involved in glycogen (glycogen synthase—GYS1) and lipid metabolism (carnitinpalmitoyl transferase—CPT1b) were determined one-day post-treatment. Additionally, phosphoenolpyruvate carboxykinase (PEPCK), pyruvate dehydrogenase 4 (PDK4), and glycogen phosphorylase (PYGM) were found to be downregulated six hours after treatment with irisin [105]. In C2C12 myotubes treated with irisin, the latter exerted a positive effect on PGC-1α, which subsequently lead to an enhancement in mitochondrial content as well as consumption of oxygen [106].

Chang et al. found a link between muscle dysfunction and irisin titers in the elderly, considering it to be a potential biomarker for sarcopenia. However, the authors used ELISA kits to determine circulating levels of the myokine [107]. Nevertheless, the relationship between muscle mass or function and irisin levels in human serum, and specifically older individuals remains a matter of debate seeing as most of the currently available detection methods lack sensitivity [104].

It has been stated that the adipokine resistin blunts human myogenesis through the activation of the NFκB pathway (a negative regulator of myogenesis), especially in obese older individuals [108]. O’Leary et al. analyzed the impact of lean and overweight subcutaneous fat conditioned media secretome on cultured muscle cells from 18–30-year-old and >65-year-old individuals. In cultured myotubes, resistin bolstered intramyocellular lipid accumulation. In addition, the adipokine influenced myotube metabolic activity by increasing both basal and maximal respiration as well as ATP production, and by boosting fatty acid oxidation [108]. Yoshiko et al. examined the relationship between the values of TNFα, adiponectin and leptin, andthe echo intensity (chosen as an indicator of muscle strength andfunction) of muscles in the upper arm, thigh, and lower back (triceps brachii, biceps femoris, rectus femoris, and multifidus) in elderly men and women [109]. The average echo intensity of the upper and lower limb muscles was negatively correlated with both circulating leptins, as well as adiponectin values [109]. Prestes et al. investigated the muscle strength responsiveness (relative muscle strength gain in 45° leg press) of olderwomen who underwent afour-month resistance training program [110]. The subjects classified as low responders demonstrated higher circulating leptin levels at baseline compared to high responders. However, a reduction of leptin and resistin plasma values was observed throughout the study in both high as well as low responders [110]. 

## 4. Age-Related Hormonal Changes and Physical Activity

**Figure 5:**
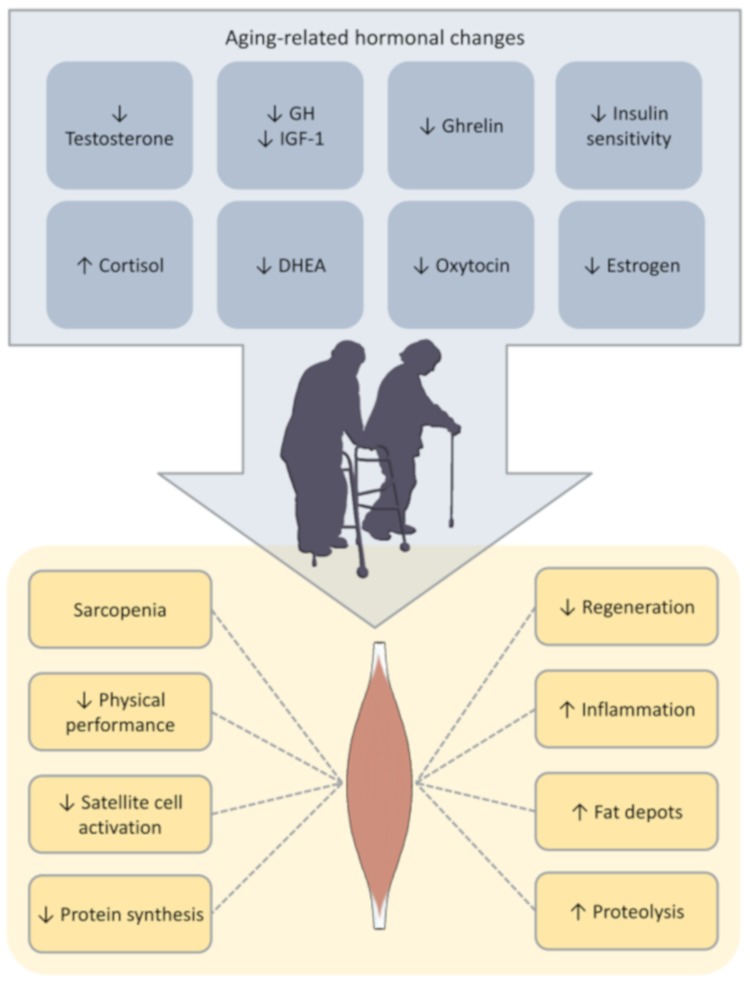
Hormonal changes and their impact on skeletal muscles.

The aging process is accompanied by variations in hormonal release (endocrine aging, Figure 5), elderly persons exhibiting lower levels of dehydroepiandrosterone (DHEA), estrogens, testosterone, growth hormone (GH), as well a dysfunctional hypothalamic-pituitary-adrenal axis which may all lead to decreased lean mass and disruptions in muscle strength and metabolism [111,112,113]. However, there is a certain amount of controversy surrounding the potential benefits of hormone replacement therapy on skeletal muscle activity in these cases [113]. Exercise has been shown to influence hormone release in elderly individuals [114]. However, the clinical relevance of these changes (specifically at the level of skeletal muscle metabolism) is yet to be fully elucidated [115,116].

Testosterone is an anabolic hormone with a pivotal role in the regulation of protein synthesis as well as regeneration at the level of skeletal muscles. In healthy men, testosterone levels suffer a 1% reduction per year, beginning from the fourth decade of life [113,114]. Nevertheless, while low testosterone levels have been associated with the risk of falls, low muscle mass and physical performance in older men, studies regarding the benefits of testosterone replacement therapy report conflicting results and notable adverse events [117,118]. Other options, such as selective androgen receptor modulators (SARMs), showed promising results with respect to the increase of skeletal muscle mass as well as the modulation of muscle metabolism (emerging as promoters of muscle anabolism) [119,120,121,122]. Physical training has been shown to enhance testosterone levels in older men. Hayes et al. measured total and free testosterone, cortisol, and sex hormone binding globulin titers in the sera of sedentary older men before and after HIIT (preceded by a conditioning phase) [115]. Cortisol levels remained fairly constant throughout the study. The total testosterone levels were significantly improved during both the conditioning stage, as well as post-HIIT, while free testosterone demonstrated a modest but statistically significant increase only after the latter [115]. Contrary to age-matched lifelong physically active individuals, sedentary older men have been shown to exhibit a notable increment of total testosterone values (but not free testosterone) and cardiorespiratory fitness following a six-week conditioning training program [123].

Nonetheless, the dynamics of testosterone release post-exercise in relation with skeletal muscle metabolism and performance remains a matter of discussion. In young male endurance athletes who underwent an 18-week intensive training program (running, circa five sessions per week), testosterone levels displayed an acute decrease (with a subgroup fulfilling androgen deficiency criteria). In contrast, preliminary findings revealed that the subjects’ physical performance was enhanced [124].

Murine studies showed that estrogen could upregulate glucose uptake by promoting the phosphorylation of certain proteins involved in the insulin signaling pathway, which, in turn, may bolster the translocation of glucose transporter 4 (GLUT4) to the muscle cell membrane [125,126,127]. In postmenopausal women, estrogen levels decrease dramatically, contributing to the decline of both muscle mass and physical performance [128]. Recent research suggests that circulating estrogen, growth hormone (GH) and dehydroepiandrosterone (DHEA) levels together with physical fitness (balance, muscle strength, and flexibility) could be raised byregular exercise in aging women. However, studies are often conducted on small sample sizes observed over short periods [129,130].

Marked disruption of the hypothalamic-pituitary-adrenal axis has been described in aging and certain chronic diseases [131,132], increased glucocorticoid levels leading to the impairment of protein synthesis and a concomitant acceleration of proteolysis at the level of skeletal muscles [133]. A number of other hormones such as insulin growth factor-1 (IGF-1), GH, and oxytocin have also been shown to impact skeletal muscle mass, function, and regeneration capacity in aging individuals [113]. Both IGF-1 and GH exert notable anabolic effects on protein metabolism [134]. Moreover, the two hormones impact skeletal muscle hypertrophy and differentiation [114]. It has been suggested that the age-related decline in IGF-1 and GH levels could play a role in the development of sarcopenia, while chronic physical exercise may boost GH-IGF-1 axis activity [114,129].

Ghrelin is an important modulator of appetite and metabolism, and aging has been associated with decreased circulating levels of the hormone [135]. Displaying potent orexigenic effects, ghrelin has also been described as a GH secretagogue hormone (endogenous ligand for the growth hormone secretagogue receptor). The 28-amino acid peptide is mainly released at the level of the stomach, but also the central nervous system, adipose tissue, and muscles [136]. Ghrelin impacts skeletal muscle metabolism, exhibiting notable anabolic effects in both humans and rodents. Research suggests that ghrelin has beneficial effects on skeletal muscle by improving function and preventing aging- or disease-associated waning/atrophy [137]. In murine models of chronic kidney disease (a condition which is frequently found in the elderly), the administration of unacylated ghrelin demonstrated beneficial effects on muscle catabolism [138].

Some authors found that ghrelin deletion in young experimental animal models did not lead to significant modifications in body composition. Following ghrelin deletion, mouse models of age-related skeletal muscle changes demonstrated a rise in the number of oxidative and fatigue-resistant type IIa muscle fibers [139]. In addition, Guillory et al. identified a relationship between ghrelin signaling and phosphorylated adenosine monophosphate-activated protein kinase (pAMPK) decline [139]. In murine models of chronic heart failure, the administration of ghrelin restored the oxidative capacity of skeletal muscle mitochondria and influenced mitochondrial biogenesis [140]. In the RESOLVE study, overweight research subjects were randomly assigned to three different physical training regimens: low-resistance–low-aerobic, low-resistance–high-aerobic and high-resistance–low-aerobic exercise [141]. Compared to baseline values, ghrelin levels were increased after three weeks and three months, respectively. The titers identified after 6 and 12 months of exercise were found to be similar to baseline values [141]. Currently, the relationship between exercise, ghrelin values, and muscle function/metabolism is yet to be fully characterized, especially in older human subjects [142].

Old age is known to be associated with a decrement in insulin sensitivity; the hormone exhibiting important anabolic effects on skeletal muscles [143]. Adiponectin has been shown to be an amplifier of insulin signaling. The adaptor protein APPL2 (leucine zipper motif isoform 2) is able to interfere with the AdipoR (adiponectin receptors 1 and 2)-APPL1 interaction involved in the adiponectin pathway [144]. Studies on murine models revealed that exercise might have beneficial effects on insulin sensitivity by diminishing the content of APPL2 in the skeletal muscles of older Fischer 344 rats [145]. Furthermore, research indicates that regular exercise may reduce the impact of aging-related insulin sensitivity in humans [146].

Bucci et al. investigated the impact of a four-month physical training program on skeletal muscle insulin sensitivity in a group of elderly women [147]. The elderly patients who were the offspring of overweight or obese mothers (OOM) demonstrated lower insulin sensitivity at the level of thigh muscles compared to the rest of the group. However, whole-body insulin sensitivity was similar to that of older women born of lean/eutrophic mothers (OLM) [147]. Resistance training improved muscle mass in both OOM and OLM, but whole-body as well as insulin sensitivity enhancement were only seen in the OOM subgroup [147]. In addition, the amelioration of insulin sensitivity post-training was more pronounced in subjects with shorter leukocyte telomere length [147]. Both exercises, as well as calorie restriction-induced weight loss, improve insulin sensitivityin older persons with excess weight. Menshikova et al. found similar improvements in insulin-stimulated glucose disposal rates at 16 weeks of either exercise or diet-induced weight loss [64]. Ha & Son also found a significant improvement in insulin sensitivity in elderly women after a 12-week aerobic and anaerobic exercise (combined) intervention [129].

Whereas aging-associated hormone imbalance and its impact on skeletal muscles continue to attract interest from researchers, extensive analysis is needed to fully decode the intricate mechanisms underlying these processes, as well as to elucidate the potential impact of physical exercise [148]. 

## 5. Inflammaging and Physical Activity

According to recent studies, aging is associated with a low-grade yet persistent accrual of such inflammatory biomarkers as IL-6, tumor necrosis factor α (TNF-α), and C-reactive protein (CRP), depicting a phenomenon widely known as inflammaging [149,150,151,152]. Inflammatory activation in older individuals has been shown to be explained by a wide variety of factors. Noteworthy, the low-grade systemic inflammation associated with advanced age does not derive from the engagement of a single signaling axis, but rather employs more complex mechanisms which may be analyzed through clusters of biomarkers. In elderly individuals, Morrisette-Thomas et al. found that TNF-α, IL-1 RA, IL-6, IL-18, high sensitivity CRP (hsCRP), as well as soluble TNF receptors I and II correlated (as a group) with mortality and chronic conditions [153]. "Garb"-aging is a term referring to the accumulation of cell/organelle-derived debris (cellular "garbage" such as formyl peptides, cardiolipin, or mitochondrial DNA) in parallel with a decreased activity of elderly individuals’ disposal mechanisms [154]. It is believed that these processes are linked to mitochondrial dysfunction and systemic inflammation in old age [154].

Systemic inflammation plays a significant role in the body composition decline of the elderly [155,156]. The metabolic disturbance associated with inflammation implies an increase in energy consumption and intense proteolysis (catabolism of skeletal muscle tissue), and may be accompanied by the extrusion of fluids into the extracellular compartment as well as by an increment of acute phase protein synthesis [157].Research showed that such pro-inflammatory cytokines as TNF-α and IL-6 display anti-myogenic properties and may contribute to the development and progression of muscle atrophy [158].

“Adaptive aging” refers to the achievement of a balance between pro- and anti-inflammatory mediators in older persons [159]. It has been stated that aging-associated low-grade systemic inflammation could be improved by diet, gut microbiota and physical exercise (particularly resistance training) [160,161,162]. Niklas et al. [163] and Tartibian et al. [164] found a significant decline of IL-6 values after exercise interventions in aging persons (walking one hour and a half per week for over 50 weeks/treadmill for 24 weeks). However, only Tartibian et al. identified lower levels of TNF-α [164]. Martins et al. described lower CRP titers after a 16-week aerobic exercise intervention [165]. Whereas the exact molecular pathways through which physical activity inflammaging-related changes in muscle metabolism, exercise has been associated with "healthy aging" [166]. Currently, there is a penury of data in this field which demands further exensive investigation [166].

## 6. Conclusions

The aging process is associated with notable changes in muscle mass, structure, and function. Importantly, aging individuals exhibit a decline in skeletal muscle metabolism, a phenomenon which may be linked to a plethora of predisposing (risk) factors such as telomere attrition, mitochondrial dysfunction, physical inactivity, hormonal changes, age-related low-grade systemic inflammation (inflammaging), as well as a hypoproteic diet (unable to counterbalance the repercussions of the age-related increase in skeletal muscle catabolism). Whereas in recent years, the subject of skeletal muscle metabolic decline in the elderly has been gathering interest amongst researchers as well as medical professionals, there are many challenges yet to be solved in order to efficiently counteract the effects of aging on muscle function.
